# Non-verbal intelligence outperforms selective attention in a visual short-term memory test

**DOI:** 10.1186/s41155-021-00200-0

**Published:** 2021-11-16

**Authors:** Luis Anunciação, Anna Portugal, Ivan Rabelo, J. Landeira-Fernandez

**Affiliations:** 1 Psychometrics and Applied Statistics, Pontifical Catholic University of Rio de Janeiro, Rio de Janeiro, RJ Brazil; 2 Psychological Assessment, Federal University of Rio de Janeiro, Rio de Janeiro, RJ Brazil; 3 Psychological Assessment, São Paulo University, São Paulo, Brazil; 4 Experimental Psychology, Pontifical Catholic University of Rio de Janeiro, Rio de Janeiro, RJ Brazil

**Keywords:** Psychometrics, Short-term memory, Intelligence, Attention, Cognitive neuroscience

## Abstract

Short-term memory is a dynamic psychological process that operates within a network in which non-verbal intelligence and attentional domains are connected. However, no consensus has been reached about which process has the greatest effect on this memory ability, which was the main objective of the present study. A sample of 1448 Brazilian participants (mean age = 26.62 years, standard deviation = 9.97 years; 53.9% females) were collectively tested on pen-and-paper standardized and validated measures of selective (ROTAS-C), alternating (ROTAS-A), and divided (ROTAS-D) attention. They also performed the R1 Non-verbal Intelligence Test and a visual short-term memory test (Memória Visual de Curto Prazo [MEMORE] test). The statistical analyses consisted of a data mining procedure, in which exhaustive automatic selection screening was performed. The results were compared with Corrected Akaike Information Criteria. The linear model met the classic assumptions of ordinary least squares and only included main effects of selective attention (standardized *β* = 0.39) and non-verbal intelligence (standardized *β* = 0.37) as main predictors (*F*_2,39_ = 7.01, *p* < 0.01, adjusted *R*^2^ = 24%). The results are discussed within a cognitive psychology framework.

Short-term memory (STM) is a vital neuropsychological process that refers to the ability to retain small amounts of information for a short period of time (Camina & Güell, 2017). Two main aspects of STM are the presence of (1) temporal decay and (2) a chunk capacity limit (Cowan, 2008). Much evidence suggests that STM is an important mediator of visual awareness, long-term phonological learning, and communication (Hambrick, Kane, & Engle, 2004; Leclercq & Majerus, 2010; Maljkovic & Martini, 2005). Accumulating evidence indicates that STM is required for almost every cognitive ability and plays a critical role in intelligence and attention (Fukuda & Vogel, 2010).

In turn, intelligence is one of the most studied psychological processes. There are many ways to conceptualize and define intelligence. Studies of intelligence were one of the landmarks of psychometric modeling in psychology. In 1904, Charles Spearman examined correlations among different ability tests and found positive intercorrelations among sets of test items. Spearman labeled this phenomenon “positive manifold” (Borg, 2018). According to Spearman, all human abilities comprise universal factors (*g*-factor) and specific factors (*s* factors). Recent findings in the literature have demonstrated the plausibility of this structure (Castejon, Perez, & Gilar, 2010; Cucina & Byle, 2017).

According to Spearman’s theory, intelligence can be viewed as an ability to efficiently adapt to the environment. Therefore, it involves learning from experience and recognizing and solving problems. Data support the stability of intelligence across the lifespan (Ramsden et al., 2011). Non-verbal intelligence refers to abstract reasoning, and its ability is partially independent of language (Anagnostou et al., 2013).

Attention is a multidimensional ability that involves the ability to focus selectively on some things while diverting focus away from others (Gazzaniga & Halpern, 2015). Its process allows attending to, filtering, and selecting among a continuous stream of information (Richard et al., 2020). Some controversies in the recent literature can be found with regard to taxonomic classifications (Posner & Boies, 1971). Nonetheless, there is growing agreement that selective, alternating, and divided attention are found in attentional processes. Selective attention refers to the ability to select and focus on particular inputs while simultaneously suppressing irrelevant or distracting information (Stevens & Bavelier, 2012). Alternating attention is frequently defined as the rapid shifting of attentional focus (Commodari, 2017). Divided attention is the ability to simultaneously produce competing responses to multiple cognitive inputs (Buchin & Mulligan, 2019; Moore Sohlberg, McLaughlin, Pavese, Heidrich, & Posner, 2000; Naveh-Benjamin, Guez, Hara, Brubaker, & Lowenschuss-Erlich, 2014).

Although evidence indicates interrelationships among these cognitive skills, the networks that underlie the structure of STM are still under debate (Engle, Laughlin, Tuholski, & Conway, 1999). Psychometric and statistical studies have explored relationships between memory and other psychological attributes but have not reached a consensus about which predictor has the greatest effect on STM (Anunciação, Portugal, Rabelo, Cruz, & Landeira-Fernandez, 2020; Richard et al., 2020). This gap in the literature impacts the theoretical building of cognitive processes and indirectly impacts clinical procedures that are related to STM.

The present study sought to determine the best predictor of visual STM, operationalized as the results of psychological tests of visual STM, attentional processes, and non-verbal intelligence. The methodological approach included a machine learning-based exploratory method that implemented a data mining algorithm.

## Methods

The present study was part of a long-term research project that integrates the psychometric and computational modeling of memory and other cognitive skills. The present study involved observational/correlational research that implemented a relational and predictive design.

### Participants

The sample comprised 1448 participants, 14–65 years of age (*M* = 26.62 years, SD = 9.97 years). Women comprised 53.9% (*n* = 781) of the sample (*χ*^2^_1_ = 8.98, *p* < 0.01), and men comprised 46.1%. With regard to level of education, 4.1% (*n* = 60) of the participants had elementary school as their highest level, 34.6% (*n* = 501) had high school as their highest level, and 61% (*n* = 883) had incomplete or complete higher education (*χ*^2^_2_ = 704.8, *p* < 0.01). Most of the participants were Brazilian (89%, *n* = 1289) from the southeast region (59.4%, *n* = 860; *χ*^2^_6_ = 2701.8, *p* < 0.01). Table 1 shows the demographics of the respondents.

**Table 1:** Demographic data of the sample

		*M*/count	*SD*/%
Age (years)		26.62	9.97
Gender	Female	781	53.9%
	Male	667	46.1%
*χ*^2^_1_ = 8.98, *p* < 0.01			
Education	Middle school	60	4.1%
	High school	501	34.6%
	Undergraduate	883	61.0%
	Not specified	4	.3%
*χ*^2^_2_ = 704.8, *p* < 0.01			
Place of birth in Brazil	Southeast Brazil	860	59.4%
	South Brazil	263	18.2%
	North Brazil	2	0.1%
	Northeast Brazil	159	11.0%
	Center-West Brazil	5	0.3%
	Foreign born	3	0.2%
	Not specified	156	10.8%
*χ*^2^_6_ = 2701.8, *p* < 0.01			
Total		1448	

*M* mean, *SD* standard deviation

χ^2^ Chi-squared test

### Procedure

Data collection occurred between 2013 and 2019. This procedure mainly relied on the voluntary participation of undergraduate and graduate students, and compulsory assessment of individuals who applied for the national driver’s license or were undergoing psychological evaluation for civilian gun ownership.

Because of Brazilian federal legislation, a psychological assessment is compulsory for everyone who applies for a driver’s license and gun ownership. This assessment is performed within 1 day. It occurs in specialized clinics or centers and should be administered by a certified psychologist. According to current legislation, psychologists administer a clinical interview and also administer psychological tests to assess non-verbal intelligence, attention and memory skills, and personality traits.

The participants in the present study were recruited from these clinics and invited to participate. All of the tests were collectively administered in pen-and-paper format. The time spent to perform a task did not last more than 30 min.

All researchers’ e-mail addresses were available to the participants if they had any questions, but no events were reported. This study was approved by the ethical committee of São Francisco University (USF), Brazil (protocol no. 0058.1.142.186-11).

### Instruments

#### Visual short-term memory test (Memória Visual de Curto Prazo [MEMORE] test)

**Fig. 1:**
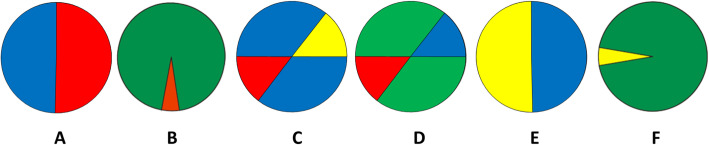
Colored circles used in the test (adapted stimuli are shown because of copyright restrictions)

The MEMORE test evaluates the ability to memorize, recall, and discriminate a previously seen stimulus and then retain it for a brief period of time. Participants are first required to memorize 12 colored circles for 1 min. After a divergent interference task that lasted about three minutes, in which the participant provided personal details on the first page of the activity, the participant was then asked to recall the 12 circles among a list of 24 circles (12 of the circles were previously seen) in any order for 2 min. No cues were presented. Figure 1 shows some of the circles that were used in the test. The test scoring procedures were based on signal detection theory, in which hits (true positive), misses (false negative), false alarms (false positive), and correct rejections (true negative) are possible. The number of false negatives (i.e., a previously presented circle was not checked) and false alarms (i.e., an absent circle was checked) are subtracted from the number of hits (i.e., a previously presented circle is checked) and correct rejections (i.e., an absent circle was not checked). The results were on a gradual ordinal scale and could vary from − 24 to + 24, in which higher scores indicated better performance.

Psychometric analysis MEMORE data suggested a one-dimensional structure (*χ*^2^_252_ = 1255.530, *p* < .001, root mean square error of approximation = 0.048, GFI = 0.860, MIREAL = 0.287), with adequate internal consistency (ordinal Cronbach’s *α* = 0.76) and high stability (test-retest correlation = 0.82). Other statistical information is available in Anunciação et al. (2020).

#### The selective (ROTAS-C), alternating (ROTAS-A), and divided (ROTAS-D) attention battery (Rabelo, Cruz, & Castro, 2020)

This pen-and-paper battery consists of three cancelation tests. All of the tasks evaluate an individual’s attentional capacity. Participants need to follow a specific pattern that is drawn on paper in 2 min. The Selective Attention Test (ROTA-C) investigates the participant’s selective attention by asking the participant to select only one target stimulus among various distracting stimuli. The Alternating Attention Route Test (ROTA-A) evaluates the participant’s ability to switch attentional focus from one stimulus to another. The Divided Attention Routes Test (ROTA-D) provides a measure of an individual’s ability to share attention, defined as the ability to mark more than one different stimulus in different ways simultaneously. The theoretical model of this task is based on Sohlberg and Mateer (Moore Sohlberg et al., 2000; Sohlberg & Mateer, 1987). The scoring of this battery is performed by subtracting the errors plus the omissions from the number of correct responses

**Fig. 2:**
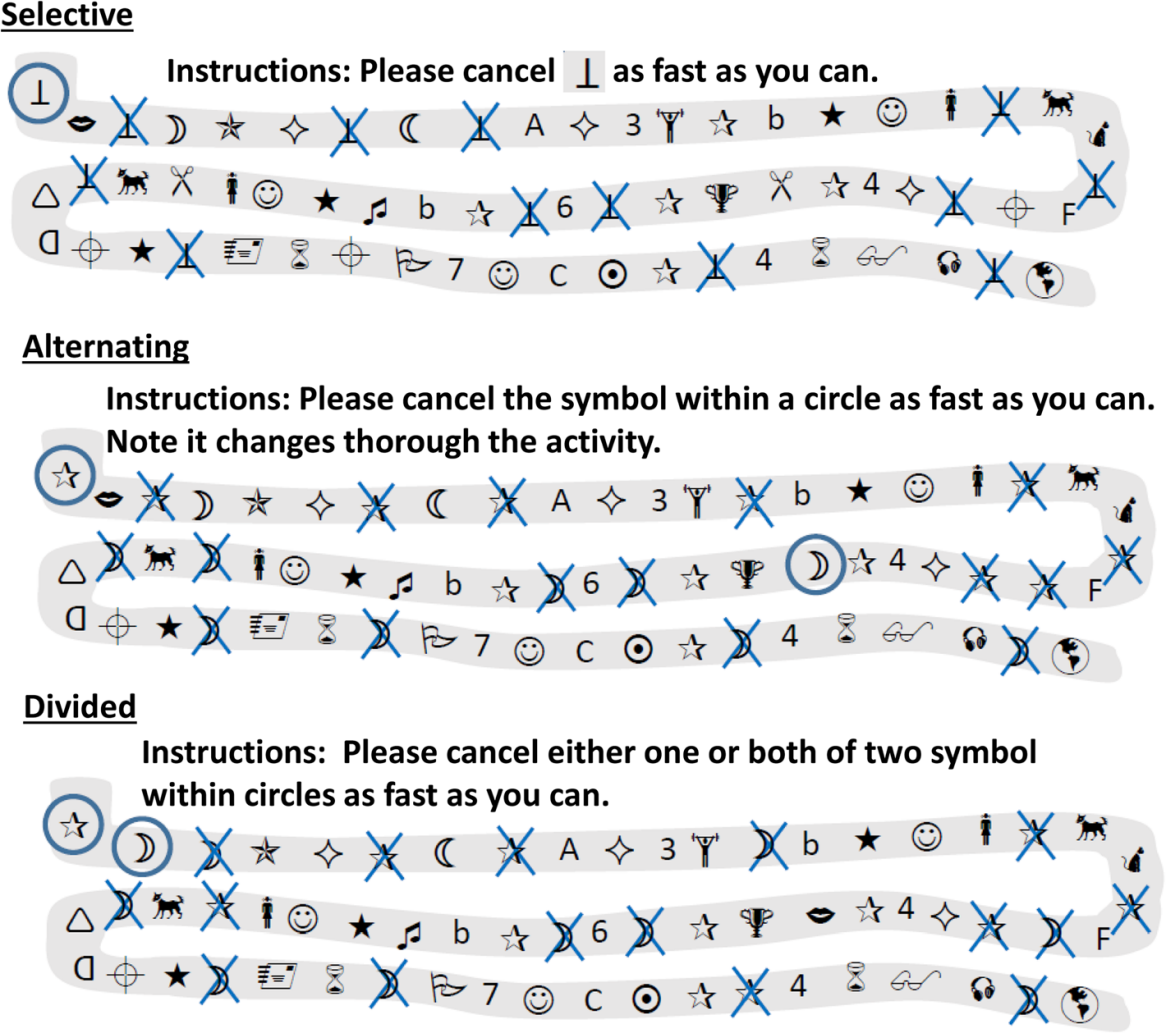
Cancelation tasks for attentional measure (adapted stimuli are shown because of copyright restrictions)

The Brazilian validation studies relied on a sample of 1251 people who were assessed between 2013 and 2018 throughout Brazil. Psychometric analyses suggested an excellent test-retest correlation (*r* = 0.9) and high internal consistency (Cronbach’s *α* = 0.80). The three domains of attention were significantly correlated (selective *vs*. alternating, *r* = 0.55; selective *vs*. divided, *r* = 0.61; alternating *vs*. divided, *r* = 0.54). A linear negative trend was found between age and performance. Older participants had lower results (*r* = 0.41). In turn, highly educated participants had higher scores than participants with only an elementary or high-school education. Females scored higher than males. Other statistical information is found in Rabelo et al. (2020). Figure 2 describes the tasks.

#### R1 Non-verbal Intelligence Test (da Silva, 2014)

**Fig. 3:**
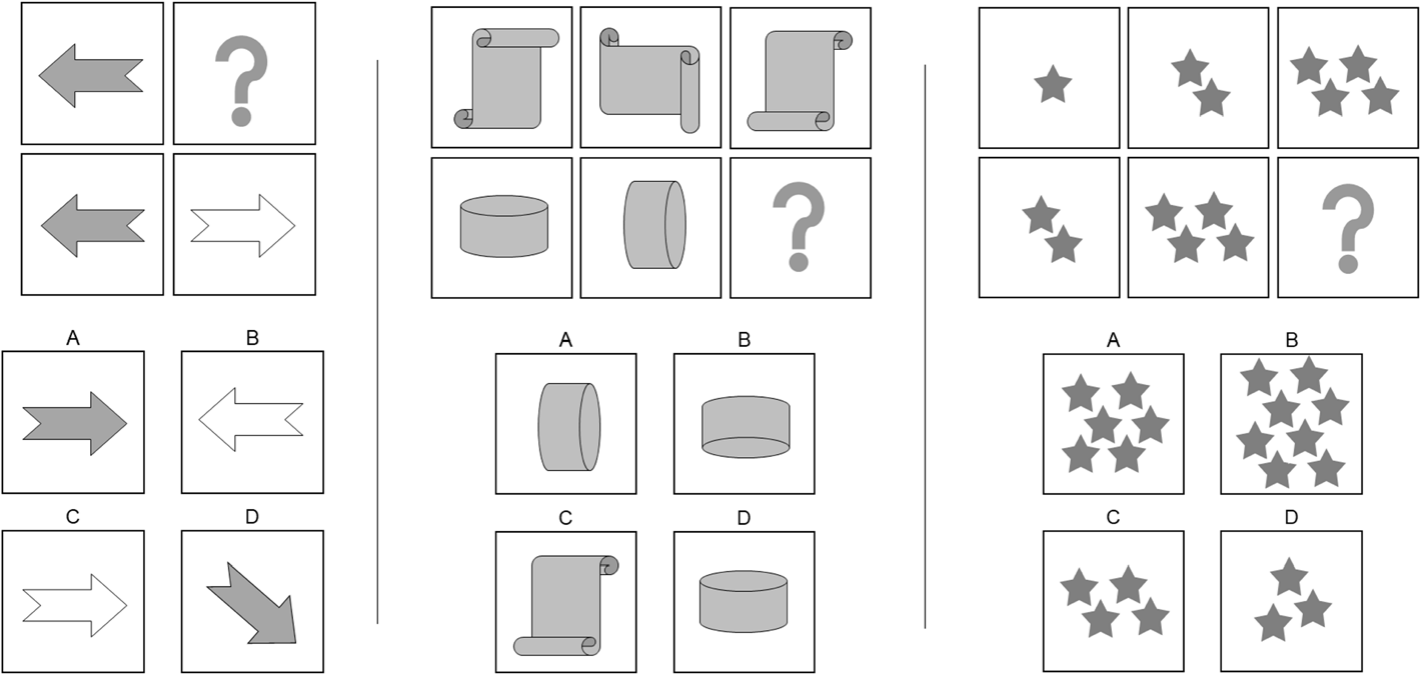
Intelligence tasks (adapted stimuli are shown because of copyright restrictions)

This is a Raven’s Matrices-based test that is widely used to measure an individual’s intellectual capacity. The task was developed based on the *g*-factor theory of intelligence and is frequently used in Brazilian specialized traffic clinics with the purpose of selecting amateur and professional drivers. The test takes approximately 30 min to complete. Figure 3 shows an approximation of the activity.

### Statistical analyses

All of the data were initially checked to ensure consistency. Outliers were not removed, and missing data were not at random and thus were not imputed. Categorical variables are expressed as frequencies and percentages. Continuous variables are expressed as the mean (*M*) and standard deviation (*SD*), quartiles, and median absolute values. In accordance with the respective user guides, the raw scores of each cognitive measure were transformed to composite scores. This transformation is described in the instrument’s subsections.

We relied on automatic selection based on Corrected Akaike Information Criteria (AICc), implemented in the glmulti package, to study the relationship between visual short-term memory (i.e., the dependent variable, considered interval-level for the purposes of data analysis) and results of the selective, alternating, and divided attention and non-verbal intelligence tasks. GLMulti heuristics involve an exhaustive screening that combines all independent variables and then compares the AICc to choose the best predictors (i.e., the model with the lowest AICc). When the tested model included an interaction term (i.e., selective attention × divided attention), the corresponding main effects were also included in accordance with the principle of “marginality” (Calcagno & de Mazancourt, 2010).

The regression assumptions were tested via visual inspection, in addition to the Shapiro-Wilk test for normality of the residuals, the Breusch-Pagan test for heteroskedasticity, and the variance inflation factor (VIF < 10) for multicollinearity.

An alpha level of .05 was used to avoid type 1 errors. The analyses were performed using R 4.0 software with the tidyverse (Wickham, 2016), glmulti (Calcagno & de Mazancourt, 2010), and olsrr (Hebbali, 2018) packages. Codes are available at https://osf.io/wyevt/

## Results

The means, *SD*s, and other statistics for the observed data for all of the instruments are presented in Table 2.

**Table 2:** Means, standard deviations, and other descriptive statistics of raw scores for all instruments

Statistics	Visual short-term memory	Alternating attention	Selective attention	Divided attention	Non-verbal intelligence
	MEMORE	ROTAS-A	ROTAS-C	ROTAS-D	R1
Mean	10.98	143.78	158.96	107.07	26.67
SD	6.26	49.77	41.82	34.49	5.32
Min	− 8	− 78.33	0	− 92.33	18
Q1	6	115	130.67	88.33	22
Median	12	146.67	157	106.67	27
Q3	16	176	185.67	123.67	30
Max	24	250	242.67	237	37
MAD	5.93	45.47	41.02	26.19	5.93

*SD* sample standard deviation, *Min* minimum value obtained, *Q1* first quartile, *Q3* third quartile, *Max* maximum value obtained, *MAD* median absolute deviation

Exhaustive screening among all possible linear models to fit the relationship between the results of the visual short-term memory [MEMORE] test and all other measures was performed using the glmulti genetic algorithm (Table 3). By default, this method uses all possible combinations, compares each computed AICc and its related weight, and then ranks the results in accordance with the lowest AICc.

**Table 3:** Top ranked predictors of visual short-term memory results

Rank	Model predictor	AICc	Weight
1	Visual memory ~ intelligence + selective attention	244.0503	0.143216
2	Visual memory ~ intelligence + selective attention + alternating attention + divided attention + alternating attention × selective attention + divided attention × selective attention	244.9526	0.091215
3	Visual memory ~ intelligence + selective attention + alternating attention + divided attention + selective attention × intelligence + alternating attention × selective attention + divided attention × selective attention	245.5923	0.066244
4	Visual memory ~ intelligence + selective attention + alternating attention + divided attention	245.6145	0.065513
5	Visual memory ~ intelligence + selective attention + alternating attention	245.9842	0.054457

~ means “is regressed on”

The algorithm computed all sets of combinations of candidates. The model fit from the best ranked models slightly favored the first (visual memory ~ intelligence + selective attention). Despite the similarity between the two well-ranked models, the first was substantially more parsimonious and included results from the non-verbal intelligence test and selective attention test (ROTAS-C; AICc = 244.05, weight = .14).

**Fig. 4:**
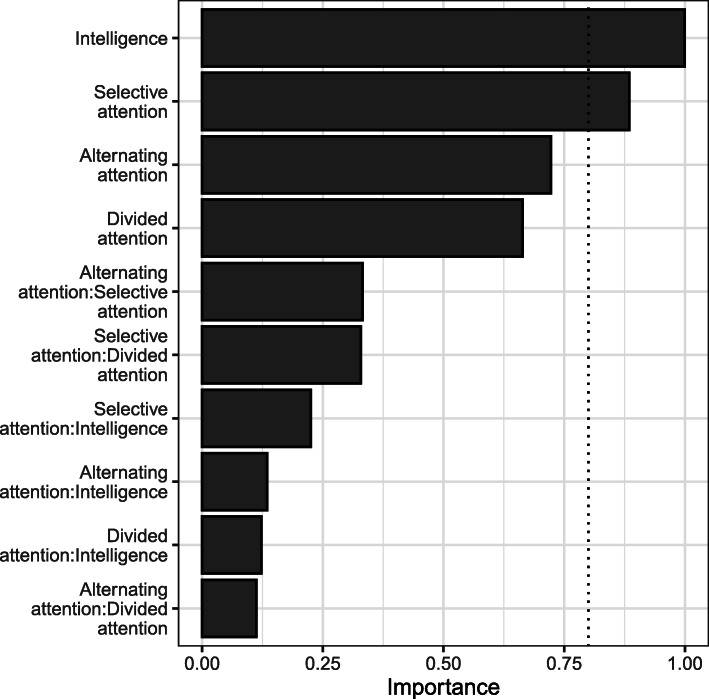
Model-averaged importance of predictors

The estimated importance value for a particular predictor is regarded as the overall support for each variable across all models in the candidate set. Because its results are equal to the sum of the weights/probabilities for the models in which the variable appears, a predictor that appears in several models with large weights will receive a high importance value. The vertical dashed line at .80 in Fig. 4 is often used as a cut-off to differentiate between the important and less important variables.

Table 4 presents the results of the final regression model, in which ~ 28% of the variance of the results of the visual short-term memory test (MEMORE) was accounted for by the predictors (*F*_2,39_ = 7.01, *p* < 0.01). The normality of the residuals was visually examined through a *Q*-*Q* plot and the Shapiro-Wilk test (*W* = .97, *p* = .6), suggesting that normality was not violated. The Breusch-Pagan test for heteroskedasticity was performed to check whether variance was constant. The results suggested homoscedasticity of the model (*p* = .15). The VIF values were < 5 (tolerance = .998), indicating that no multicollinearity was present.

**Table 4:** Final multiple linear regression model

	Unstandardized results		Standardized results	Inferential statistics		Confidence interval	
Model	*b*	Standard error	*Β*	*t*	*p*	Lower	Upper
Intercept	− 9.75	5.704		− 1.709	.096	− 21.307	1.806
Non-verbal intelligence test (R1)	.388	.139	.39	2.786	.008	.106	.671
Selective attention (ROTAS-C)	.073	.028	.369	2.63	.012	.017	.129
*R*^*2*^ .275							
Adjusted *R*^2^ .236							
Root Mean Square Error 4.742							

The standardized coefficient enables comparisons of the effect size of each predictor. A larger value denotes a greater effect on the dependent variable.

## **Discussion**

Short-term memory is one of the most important neuropsychological processes. The maintenance of information over a short period of time is needed in almost every cognitive process. However, only a few studies have investigated the role of attention and intelligence in visual short-term memory ability, especially in non-clinical samples. The present study took advantage of a series of psychometric validation studies of cognitive measures that are used in the context of compulsory psychological assessment. Our results indicated that the unique contributions of selective attention and non-verbal intelligence are the best candidates over a set of different models.

Previous studies established a relationship between memory and other cognitive skills, such as intelligence and attention (Engle et al., 1999; Veer, Luyten, Mulder, van Tuijl, & Sleegers, 2017; Zanto & Gazzaley, 2009). Non-verbal intelligence and selective attention are both crucial constructs that are used to predict performance on various complex cognitive tasks. The present results indicated that intelligence had a greater effect size, which was partially consistent with the findings of Alosco et al. (2014), Fenn and Hambrick (2015), Miller, Spitznagel, Hughes, Rosneck, and Gunstad (2018), and Haavisto and Lehto (2005), although the first studies were performed with clinical samples, and the latter studies relied on data from Air Force recruits. In our study, we also used validated measures in which the sample size was equal or larger than the original studies.

Alternating and divided attention were shown to be predictors that were not selected from the data mining procedure. This result can be justified by previous neuroscientific studies. Although there are close links between attentional mechanisms, alternating and divided attention are known to disrupt memory encoding (Buchin & Mulligan, 2019; Naveh-Benjamin et al., 2014).

Considering the previous findings, this outcome corroborates the multidimensionality of attentional processes. Therefore, although correlations between attentional abilities are substantial, their effects on STM are dissimilar, stressing differences between these aspects of attention.

These outcomes are not universal or free of academic dispute. Other findings suggest that selective attention can be a moderator of the relationship between intelligence and short-term memory (Unsworth & Engle, 2005). These authors proposed that directed attention assumes a pivotal role in orienting memory maintenance (Oren et al., 2016) and activating higher-order visual areas that are needed to process stimuli (Lepsien, Thornton, & Nobre, 2011).

When focusing on intelligence as a predictor of visual short-term memory, a partial explanation can be provided by the *g*-factor theory. Evidence from several research fields indicates that the *g*-factor has high explanatory power in a wide range of dimensions of behavior and across diverse mental tasks (Kärner, 2017). Thus, higher intelligence can lead to better strategies during the encoding phase of the short-term memory process, improve memory storage capacity, and enhance mental speed that is needed to perform memory tasks (Colom, Abad, Quiroga, Shih, & Flores-Mendoza, 2008).

The present study had two characteristics that limited a deeper understanding of the interplay with the broader literature. First, our results were based on psychometric modeling and thus were gathered from cognitive tests that were designed to operationally analyze a particular theory. The cognitive mechanisms that underlie the relationships among psychological constructs are still unknown, and multiple and competing definitions of the same constructs can be found (Jurado & Rosselli, 2007). As often reported in psychology, slight changes in the way one conceptualizes and measures these constructs tend to lead to substantial differences (Sijtsma, 2012). Thus, the results of the previous studies on which we relied in the present study may also reveal theoretical assumptions that are different from our assumptions, thereby limiting the conclusions that can be drawn. However, we stress that this limitation is frequently found in psychometric studies.

We consider that visual short-term memory is still available after 1–3 min, which is a known attribute of tests that are used among clinical neuropsychologists and neurologists in screening procedures (Buschke et al., 1999; Kuslansky, Buschke, Katz, Sliwinski, & Lipton, 2002). However, this contrasts with some assertions that the duration of short-term memory is only 15–30 s when no form of memory rehearsal is present (Alvarez & Cavanagh, 2004; Jiang, Olson, & Chun, 2000). Moreover, definitions that derive from multiple attention models in the literature, in which selective attention is considered to involve the maintenance of a cognitive/behavioral set despite competing stimuli, are not necessarily consensual (Sohlberg & Mateer, 1987; Stevens & Bavelier, 2012).

The statistical approach that was used to guide the results relied on a computer-aided algorithm, based on the notion of parsimony that is achieved by a lower AICc. Parsimony argues that a model with fewer predictors is preferred over a model with numerous predictors if the latter yields a nonsignificant, negligible, or trivial increase in explanatory power. Despite the use of the AICc to define the model that fit the data best and that is typically implemented in linear models (Calcagno & de Mazancourt, 2010; Hasan Örkcü, 2013), the specific criterion that is applied by the researcher is arbitrary. In the fields of psychology and other empirical sciences, this is still an open research question, and many argue that these models can give markedly different results based on the same data. We are also aware that statistical models help the understanding of psychological processes, with varying degrees of fit from reality.

However, these issues are not particular features of only the present study and instead are a frequent condition in other psychometric endeavors (Sijtsma, 2012). Thus, the instruments that were used in the present study have sound psychometric studies to minimize these limitations (including the clinical utility), and the automatic procedure exhaustively tested all predictor combinations (Calcagno & de Mazancourt, 2010).

The present results shed further light on cognitive processes and may be of interest especially to cognitive psychologists and neuropsychologists. Unveiling the best predictors of STM could aid the theoretical building of cognitive processes and indirectly impact clinical procedures that are related to STM, which plays an important role in psychological processing.

## Conclusion

The present study sought to integrate psychometric and statistical modeling to identify and compare effect sizes of results from attentional and non-verbal intelligence tests on the results of a visual short-term memory test. Despite the methodological challenges of studying relationships between latent variables, computer-aided model selection was based on the lower value of the AICc, suggesting that the main effects of non-verbal intelligence and selective attention were the best predictors of visual short-term memory results.

The effect size of non-verbal intelligence was higher than the effect size of selective attention when both processes were compared. Alternating and divided attention were also explored, but they were not significantly related to the outcome. Further research is needed to determine the reliability and generalization of these findings.

## Data Availability

The datasets generated and/or analyzed during the current study are available in the Open Science Framework repository, at https://osf.io/wyevt/

## References

[CR1] Alosco ML, Garcia S, Spitznagel MB, van Dulmen M, Cohen R, Sweet LH, Josephson R, Hughes J, Rosneck J, Gunstad J (2014). Cognitive performance in older adults with stable heart failure: Longitudinal evidence for stability and improvement. Aging, Neuropsychology, and Cognition.

[CR2] Alvarez GA, Cavanagh P (2004). The capacity of visual short-term memory is set both by visual information load and by number of objects. Psychological Science.

[CR3] Anagnostou E, Mankad D, Diehl J, Lord C, Butler S, McDuffie A, Shull L, Ashbaugh K, Koegel RL, Volkmar FR, Naples A, Doggett R, Koegel RL, Hooper SR, Casanova M, Hoffman EJ, McFadden K, Anderson GM, Gupta AR (2013). Nonverbal intelligence. Encyclopedia of autism spectrum disorders.

[CR4] Anunciação L, Portugal AC, Rabelo IS, Cruz RM, Landeira-Fernandez J (2020). Propriedades psicométricas de instrumento de Memória Visual de Curto Prazo (MEMORE). Revista Neuropsicologia Latinoamericana.

[CR5] Borg I (2018). A note on the positive manifold hypothesis. Personality and Individual Differences.

[CR6] Buchin ZL, Mulligan NW (2019). Divided attention and the encoding effects of retrieval. Quarterly Journal of Experimental Psychology.

[CR7] Buschke H, Kuslansky G, Katz M, Stewart WF, Sliwinski MJ, Eckholdt HM, Lipton RB (1999). Screening for dementia with the Memory Impairment Screen. Neurology.

[CR8] Calcagno, V., & de Mazancourt, C. (2010). glmulti: An R package for easy automated model selection with (generalized) linear models. Journal of Statistical Software. 10.18637/jss.v034.i12

[CR9] Camina, E., & Güell, F. (2017). The neuroanatomical, neurophysiological and psychological basis of memory: Current models and their origins. *Frontiers in Pharmacology*, *8*10.3389/fphar.2017.00438. 10.3389/fphar.2017.00438 PMC5491610 28713278

[CR10] Castejon JL, Perez AM, Gilar R (2010). Confirmatory factor analysis of Project Spectrum activities. A second-order g factor or multiple intelligences?. Intelligence.

[CR11] Colom R, Abad FJ, Quiroga MÁ, Shih PC, Flores-Mendoza C (2008). Working memory and intelligence are highly related constructs, but why?. Intelligence.

[CR12] Commodari E (2017). Novice readers: The role of focused, selective, distributed and alternating attention at the first year of the academic curriculum. I-Perception.

[CR13] Cowan, N. (2008). Chapter 20 What are the differences between long-term, short-term, and working memory? (pp. 323–338). 10.1016/S0079-6123(07)00020-9 10.1016/S0079-6123(07)00020-9 PMC2657600 18394484

[CR14] Cucina J, Byle K (2017). The bifactor model fits better than the higher-order model in more than 90% of comparisons for mental abilities test batteries. Journal of Intelligence.

[CR15] Engle RW, Laughlin JE, Tuholski SW, Conway ARA (1999). Working memory, short-term memory, and general fluid intelligence: A latent-variable approach. Journal of Experimental Psychology: General..

[CR16] Fenn KM, Hambrick DZ (2015). General intelligence predicts memory change across sleep. Psychonomic Bulletin & Review.

[CR17] Fukuda K, Vogel EK (2010). Visual short term memory serves as a gateway to long term memory. Journal of Vision.

[CR18] Gazzaniga, M. S., & Halpern, D. F. (2015). *Psychological science (fifth edition)*. W. W. Norton & Company.

[CR19] Haavisto M-L, Lehto JE (2005). Fluid/spatial and crystallized intelligence in relation to domain-specific working memory: A latent-variable approach. Learning and Individual Differences.

[CR20] Hambrick, D. Z., Kane, M. J., & Engle, R. W. (2004). The role of working memory in higher-level cognition: Domain-specific versus domain-general perspectives. In *Cognition and Intelligence*, (pp. 104–121). Cambridge University Press 10.1017/CBO9780511607073.007.

[CR21] Hasan Örkcü, H. (2013). Subset selection in multiple linear regression models: a hybrid of genetic and simulated annealing algorithms. *Applied Mathematics and Computation*, *219*(23), 11018–11028 10.1016/j.amc.2013.05.016.

[CR22] Hebbali, A. (2018). olsrr: Tools for building OLS regression models. R package version 0.5.2. https://cran.r-project.org/package=olsrr

[CR23] Jiang Y, Olson IR, Chun MM (2000). Organization of visual short-term memory. Journal of Experimental Psychology: Learning, Memory, and Cognition.

[CR24] Jurado MB, Rosselli M (2007). The elusive nature of executive functions: A review of our current understanding. Neuropsychology Review.

[CR25] Kärner T (2017). A mixed-methods study of physiological reactivity to domain-specific problem solving: Methodological perspectives for process-accompanying research in VET. Empirical Research in Vocational Education and Training.

[CR26] Kuslansky G, Buschke H, Katz M, Sliwinski M, Lipton RB (2002). Screening for Alzheimer’s disease: The memory impairment screen versus the conventional three-word memory test. Journal of the American Geriatrics Society.

[CR27] Leclercq A-L, Majerus S (2010). Serial-order short-term memory predicts vocabulary development: Evidence from a longitudinal study. Developmental Psychology.

[CR28] Lepsien J, Thornton I, Nobre AC (2011). Modulation of working-memory maintenance by directed attention. Neuropsychologia.

[CR29] Maljkovic V, Martini P (2005). Implicit short-term memory and event frequency effects in visual search. Vision Research.

[CR30] Miller LA, Spitznagel J, Hughes J, Rosneck G, Gunstad J (2018). Final Program Forty Sixth Annual Meeting International Neuropsychological Society. Journal of the International Neuropsychological Society.

[CR31] Moore Sohlberg, M., McLaughlin, K. A., Pavese, A., Heidrich, A., & Posner, M. I. (2000). Evaluation of attention process training and brain injury education in persons with acquired brain injury. Journal of Clinical and Experimental Neuropsychology, 22(5), 656–676. 10.1076/1380-3395(200010)22:5;1-9;FT656 10.1076/1380-3395(200010)22:5;1-9;FT656 11094401

[CR32] Naveh-Benjamin M, Guez J, Hara Y, Brubaker MS, Lowenschuss-Erlich I (2014). The effects of divided attention on encoding processes under incidental and intentional learning instructions: Underlying mechanisms?. Quarterly Journal of Experimental Psychology.

[CR33] Oren, N., Shapira-Lichter, I., Lerner, Y., Tarrasch, R., Hendler, T., Giladi, N., & Ash, E. L. (2016). How attention modulates encoding of dynamic stimuli. *Frontiers in Human Neuroscience*, *10*10.3389/fnhum.2016.00507. 10.3389/fnhum.2016.00507 PMC5073125 27818628

[CR34] Posner MI, Boies SJ (1971). Components of attention. Psychological Review.

[CR35] Rabelo IS, Cruz R, Castro NR (2020). Bateria Rotas de Atenção: Rota da Atenção Concentrada (ROTA C), Rota da Atenção Dividida (ROTA D) e Rota da Atenção Alternada (Rota A).

[CR36] Ramsden S, Richardson FM, Josse G, Thomas MSC, Ellis C, Shakeshaft C, Seghier ML, Price CJ (2011). Verbal and non-verbal intelligence changes in the teenage brain. Nature.

[CR37] Richard G, Petersen A, Ulrichsen KM, Kolskår KK, Alnæs D, Sanders A-M, Dørum ES, Ihle-Hansen H, Nordvik JE, Westlye LT (2020). TVA-based modeling of short-term memory capacity, speed of processing and perceptual threshold in chronic stroke patients undergoing cognitive training: Case-control differences, reliability, and associations with cognitive performance. PeerJ.

[CR38] Sijtsma K (2012). Psychological measurement between physics and statistics. Theory & Psychology.

[CR39] da Silva MA (2014). Estudos sobre a dimensionalidade do R-1: Teste não verbal de inteligência.

[CR40] Sohlberg MM, Mateer CA (1987). Effectiveness of an attention-training program. Journal of Clinical and Experimental Neuropsychology.

[CR41] Stevens C, Bavelier D (2012). The role of selective attention on academic foundations: A cognitive neuroscience perspective. Developmental Cognitive Neuroscience.

[CR42] Unsworth N, Engle R (2005). Working memory capacity and fluid abilities: Examining the correlation between Operation Span and Raven. Intelligence.

[CR43] Veer IM, Luyten H, Mulder H, van Tuijl C, Sleegers PJC (2017). Selective attention relates to the development of executive functions in 2,5- to 3-year-olds: A longitudinal study. Early Childhood Research Quarterly.

[CR44] Wickham, H. (2016). tidyverse: Easily install and load “Tidyverse” packages. In R package version 1.0.0. https://cran.r-project.org/package=tidyverse

[CR45] Zanto TP, Gazzaley A (2009). Neural suppression of irrelevant information underlies optimal working memory performance. Journal of Neuroscience.

